# Zinc(II) Complexes with Amino Acids for Potential Use in Dermatology: Synthesis, Crystal Structures, and Antibacterial Activity

**DOI:** 10.3390/molecules25040951

**Published:** 2020-02-20

**Authors:** Michał Abendrot, Lilianna Chęcińska, Joachim Kusz, Katarzyna Lisowska, Katarzyna Zawadzka, Aleksandra Felczak, Urszula Kalinowska-Lis

**Affiliations:** 1 Department of Cosmetic Raw Materials Chemistry, Faculty of Pharmacy, Medical University of Lodz, Muszyńskiego 1, 90-151 Łódź, Poland; michal.abendrot@gmail.com; 2 Faculty of Chemistry, University of Lodz, Pomorska 163/165, 90-236 Łódź, Poland; 3 Institute of Physics, University of Silesia, 75 Pułku Piechoty 1, 41-500 Chorzów, Poland; joachim.kusz@us.edu.pl; 4 Department of Industrial Microbiology and Biotechnology, Faculty of Biology and Environmental Protection, University of Lodz, 12/16 Banacha Street, 90-237 Łódź, Poland; katarzyna.lisowska@biol.uni.lodz.pl (K.L.); katarzyna.zawadzka@biol.uni.lodz.pl (K.Z.)

**Keywords:** zinc(II) complexes, proteinogenic amino acids, crystal structures, antibacterial activity, Hirshfeld surface analysis

## Abstract

The multifunctional profile of Zn^2+^ has influenced its great popularity in various pharmaceutical, food, and cosmetic products. Despite the use of different inorganic and organic zinc derivatives, the search for new zinc-containing compounds with a safer skin profile still remains an open issue. The present paper describes the synthesis, structural characterization, and antibacterial activity of zinc(II) complexes with proteinogenic amino acids as potential candidates for dermatological treatments. The obtained complexes are of the general formula [Zn(AA)_2_], where AA represents an amino acid (L-Glu, Gly, L-His, L-Pro, L-Met, and L-Trp). Their synthesis was designed in such a way that the final bis(aminoacidate) zinc(II) complexes did not contain any counter-ions such as Cl^−^, NO_3_^−^, or SO_4_^2−^ that can cause some skin irritations. The chemical structure and composition of the compounds were identified by ^1^H NMR spectroscopy and elemental analysis, and four were also characterized by single-crystal X-ray diffraction. The Hirshfeld surface analysis for the Zn^2+^ metallic center helped to determine its coordination number and geometry for each complex. Finally, the antibacterial properties of the complexes were determined with respect to three Gram-positive strains, viz. *Staphylococcus aureus* ATCC 6538, *Staphylococcus epidermidis* ATCC 12228, and *Streptococcus pyogenes* ATCC 19615, and two Gram-negative bacteria, viz. *Escherichia coli* ATCC 25992 and *Pseudomonas aeruginosa* ATCC 27853, and were compared with the activity of zinc 2-pirrolidone 5-carboxylate (ZnPCA), commonly applied in dermatology. It was found that the Zn(II) complexes with methionine and glycine exhibited a higher antibacterial activity than the tested standard, and the antimicrobial properties of complex with Trp were satisfactory. The results of the antimicrobial activity examination allow us to postulate that the obtained zinc complexes might become new active substances for use in dermatological products.

## 1. Introduction

Zinc derivatives are often utilized in pharmaceutical, food, and cosmetic products. Their popularity in medicine is associated with the antimicrobial, antifungal, antioxidant, and anti-inflammatory properties of Zn^2+^ [1]. Zinc is considered as one of the most important trace elements in the human body, and its coordination chemistry is of considerable interest. It is incorporated in the active center of many essential enzymes, such as superoxide dismutase (SOD), carbonic anhydrase, carboxypeptidase, and alcohol dehydrogenase [2,3]. It plays a vital role in sustaining proper human body function through bone formation, cell-mediated immunity, brain function, and tissue growth, among others [4,5]. Zinc has the ability to accept certain proteins domains to form zinc-finger proteins (ZNFs): these can interact with DNA, RNA, and proteins such as PAR, and are involved in the regulation of several cellular processes [6]. It has greater acceptor affinity than calcium (Ca^2+^) or magnesium (Mg^2+^) [7]. Zn^2+^ can create inert chelate complexes by accepting the free electron pairs of O-, N-, S- from certain amino acid motifs. Not only it does play a crucial role in a variety of biochemical processes, but it can also participate in numerous reactions in organometallic chemistry. Although zinc ions typically have a coordination number of 4, they can also form trigonal bipyramidal and octahedral complexes [8,9].

Several zinc derivatives are used in dermatology, the most common of which are zinc sulphates. However, they have been found to possess low bioavailability and can irritate the skin. Thus, there is a great interest in identifying safer alternatives with superior bioavailability [1]. One potential group of candidates comprises zinc compounds containing organic moieties, which in general have shown better tolerability and bioavailability than the inorganic salts [10,11,12]. The exact organic ligand structure finally determines its main direction of use. A great example is zinc ricinoleate, which does not possess bactericidal properties but serves as an anti-odor agent. Another organic derivative, zinc pyrithione, demonstrates antifungal activity against *Pityrosporum* (*Malassezia*) and exhibits an antibacterial activity, whereas zinc stearate is known for its emulsion-stabilizing properties and lack of antimicrobial properties [1]. Although many organic zinc derivatives are utilized in dermatological products, zinc-containing antimicrobial agents are becoming insufficient due to growing bacterial resistance. That is why the search for newer zinc antimicrobials still remains a big challenge. Interestingly, there are a few zinc proteinogenic amino acids complexes which are widely used in dietary supplements and animal feed, such as zinc bisglycinate and zinc methionine, but their use in dermatological products is rare [13,14,15]. It seems that conducting research in this direction could be very promising. Although the effect of zinc derivatives ultimately depends on their administered concentration, zinc compounds are generally recognized as safe in trace amounts [16]. Taking into consideration that L-α-amino acids are biogenic molecules, their zinc complexes should reveal an excellent safety profile which is worth examining.

Zinc oxide, zinc gluconate, zinc pyrithione, and zinc 2-pirrolidone 5-carboxylate (ZnPCA) are also common ingredients in sunscreens and anti-acne and anti-dandruff agents [17,18,19,20]. However, to overcome drug resistance, new antimicrobial agents are always required for dermatological disorders, especially for acne vulgaris. A useful alternative to the zinc derivatives in current use may be zinc complexes of proteinogenic amino acids: their good safety profile, high bioavailability [11,21], and potential antioxidant activity [22,23], as well as the antimicrobial properties of Zn^2+^ [21], make them particularly well suited for use in both oral preparations and dermal products. Previous studies have examined the antimicrobial activity of various complexes of transition metals, including zinc, with amino acid ligands: for example with L-alanine- and L-aspartic acid-capped ZnS nanoparticles [24] and mixed complexes with L-proline and kojic acid [21].

**Scheme 1:**
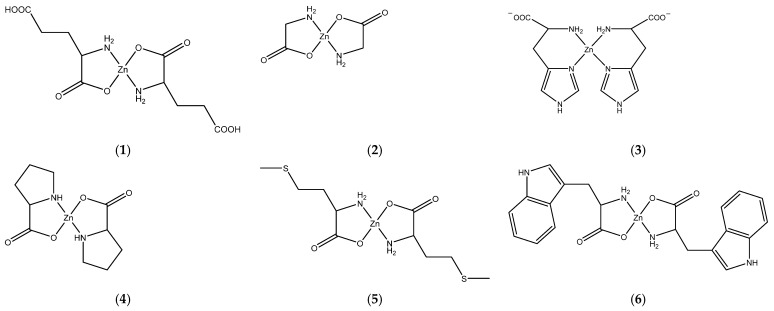
Chemical structures of Zn(II) complexes with L-glutamic acid (**1**), glycine (**2**), L-histidine (**3**), L-proline (**4**), L-methionine (**5**), and L-tryptophan (**6**).

The main aim of the present study is to examine innovative zinc derivatives with potential use in dermatology and also in the cosmetic and pharmaceutical industries. The antimicrobial activity of bis(aminoacidate) zinc(II) complexes without an anionic ingredient has not yet been examined. Theoretically, these derivatives should be better absorbed and safer due to their nonionic structure and weak influence on the skin pH which will make them excellent ingredients for dermal products. The paper describes the synthesis and full characterization of novel zinc coordination compounds with selected amino acids (AAs), viz. L-Glu, Gly, L-His, L-Pro, L-Met, and L-Trp (Scheme 1), and evaluates their antibacterial activity on three Gram-positive strains, viz. *Staphylococcus aureus* ATCC 6538, *Staphylococcus epidermidis* ATCC 12,228, and *Streptococcus pyogenes* ATCC 19615, and two Gram- negative bacteria, viz. *Escherichia coli* ATCC 25992 and *Pseudomonas aeruginosa* ATCC 27853. Bacterial strains selected for the present studies are microorganisms that cause various disorders from urinary, respiratory, and heart diseases to skin and wound infections. *Staphylococcus aureus* is a component of human natural microflora, but under certain conditions it can colonize skin and soft tissues, causing infection and contributing to atopic dermatitis or hand eczema [25,26]. *Staphylococcus epidermidis* and *Streptococcus pyogenes* are often isolated from wounds and skin lesions [27]. Also, *Pseudomonas aeruginosa* can cause mild and severe skin and soft tissue infections [28]. It is worth mentioning that infections of wounds, burns, and skin are particularly difficult to treat, especially if they are caused by multi-resistant microorganisms. Therefore, the search for new effective drugs is extremely important.

## 2. Results and Discussion

### 2.1. Synthesis and Characterization of Zn(II) Complexes **1–6**

The synthesized zinc(II) complexes were prepared using two different simple methods, with compounds **1** to **4** produced by reacting the corresponding amino acid with an aqueous suspension of zinc hydroxide (Method I), and compounds **5** and **6** by reacting a sodium salt of the corresponding amino acid with zinc(II) chloride in water (Method II). The choice of the method was related to the properties of the compounds. It is worth noting each method was performed to avoid the presence of counter-ions, such as Cl^−^ or NO_3_^−^, in the final zinc(II) complexes. All synthesized complexes have the general formula [Zn(AA)_2_], with AA representing an amino acid: L-Glu, Gly, L-His, L-Pro, L-Met, and L-Trp.

Regarding Method I, the water-soluble zinc(II) complexes containing L-Glu, Gly, L-His, and L-Pro amino acids (**1**–**4**), were synthesized according to a general procedure described to obtain a Zn(II) complex with methionine [25]. The method is based on the addition of an aqueous solution of an appropriate amino acid (AA) to an aqueous suspension of zinc hydroxide at a molar ratio of 2:1. Method I:

Zn(OH)_2 (susp.)_ + 2 AA → [Zn(AA)_2_] + 2 H_2_O AA = Glu, Gly, His, and Pro(1)

Zinc hydroxide Zn(OH)_2_ was firstly obtained by reacting zinc(II) chloride with 1M NaOH at a molar ratio of 1 to 2.

The suspension of Zn(OH)_2_ disappeared completely during the synthesis of Zn(II) complexes **1** to **4**, i.e., those with Glu, Gly, His, and Pro. The resulting water-soluble complexes were isolated from the reaction solutions, either by partial concentration on a rotary evaporator or by allowing the solvent to evaporate slowly at room temperature.

Regarding Method II, the Zn(II) complexes of Met and Trp (**5**–**6**) were synthesized as follows:

ZnCl_2_ + 2 (AA)Na→ [Zn(AA)_2_] + 2 NaCl AA = Met and Trp(2)

The process was preceded by an amino acid alkalization step.

Both methods were used to prepare the poorly water-soluble compound **5**, i.e., Zn(II) with methionine. Although the obtained products had the same structure, method II gave a higher yield. The complex obtained by method I was characterized by X-ray and elemental analysis [29].

Method II, starting with ZnCl_2_ and AA sodium salt, was not suitable for the synthesis of water-soluble complexes (**1** to **4**), because it would be difficult to remove the water-soluble by-product, NaCl. However, method II could be used to obtain Zn(II) complexes **5** and **6**. These compounds were precipitated directly from the reaction mixtures and were easily isolated from the water-soluble by-product.

Although the synthesis of [Zn(Pro)2] (**4**) has been already described in the literature [30], the strategy was different from the one we used. It was performed by reacting zinc acetate with proline in the presence of trimethylamine, using methanol as a solvent. The compound obtained in this way was used as a catalyst for some organic transformations.

Zinc(II) complexes (**1**–**6**) were characterized by elemental analysis and ^1^H NMR spectroscopy. Four of them, containing Gly, Pro, His, and Met ligands, were also characterized by X-ray single-crystal crystallography. All but one of the obtained zinc (II) complexes constitute five-membered chelates, with each amino moiety coordinated to the zinc ion through the nitrogen atom and the oxygen atom of the deprotonated carboxyl group. The Zn-histidine complex, in contrast, forms a six-membered chelate ring through coordination with two N atoms of the histidine moiety, viz. the -NH_2_ group and the N of the imidazole ring. None of the formed complexes contain any counter-ions; it is in accordance with the synthesis process of Zn^2+^ cations with amino acids with a deprotonated carboxyl group.

The ^1^H NMR spectra of Zn(II) complexes and free ligands were recorded in D_2_O (**1**–**5**) or DMSO (**6**). When using D_2_O, no signals of amino and carboxyl protons nor protons connected with nitrogen atoms of heterocyclic rings were identified. These protons were exchanged for deuterium in solutions. Thus, the spectra in D_2_O solvent illustrate the image of protons associated with carbon atoms. The signals of the protons for CH_2_, CH of heterocyclic rings, or CH-N of zinc complexes were more or less shifted compared to these of the free amino acids (Appendix S1). The most significant shifts, amounting to 0.12, 0.30, 0.14, 0.12, 0.20, and 0.11 ppm, were respectively observed for CH_2_(γ) (**1**), CH_2_ (**2**), CH(5′) (**4**), CH(2) (**4**), CH(α) (**5**), and CH_2_(β) (**6**) protons.

### 2.2. Crystal and Molecular Structures of Compounds **2–5**

The crystallographic data obtained from the structural analyses of (**2**), (**3**), (**4**), and (**5**) have been deposited with the Cambridge Crystallographic Data Centre (CCDC Nos. 1966206-1966209). These data can be obtained free of charge from CCDC via www.ccdc.cam.ac.uk/data_request/cif. Basic information pertaining to crystal parameters and structure refinement is summarized in Table 1.

#### 2.2.1. A Single-Crystal Structure of **2**

**Figure 1:**
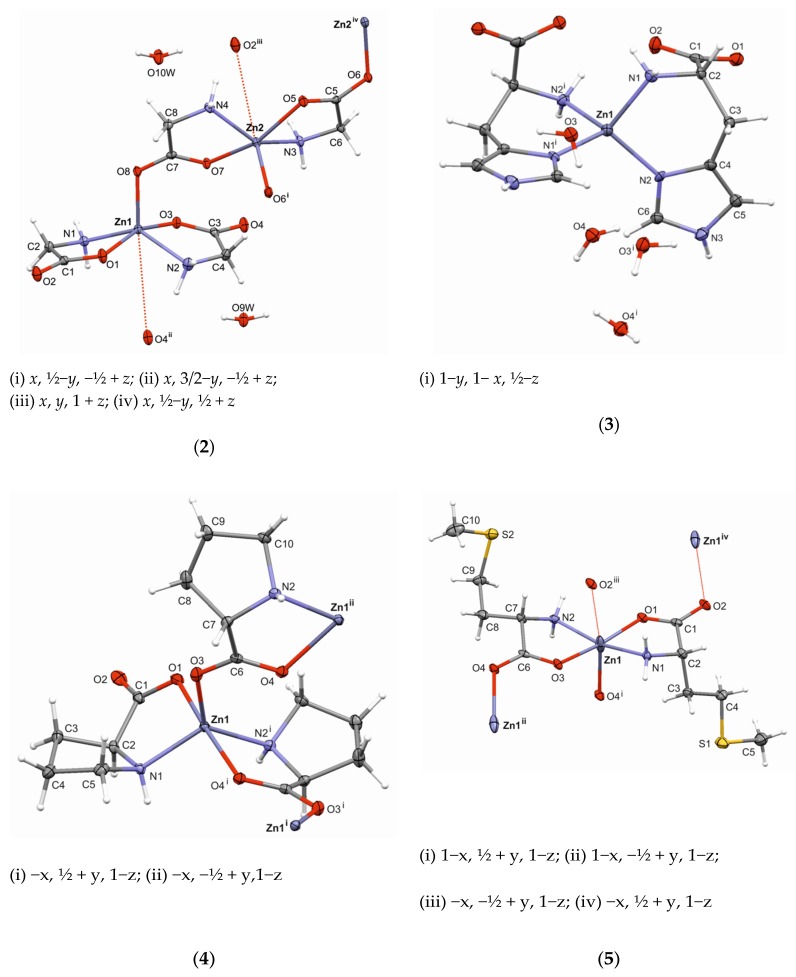
The molecular structures of Zn(II) complexes with glycine (**2**), L-histidine (**3**), L-proline (**4**), and L-methionine (**5**), showing the atom-numbering scheme. Displacement ellipsoids are drawn at the 50% probability level and hydrogen atoms are shown as small spheres of arbitrary radii.

As shown in Figure 1, the asymmetric unit of (**2**) consists of four independent glycine moieties as monoanions (Gly-1, Gly-2, Gly-3, Gly-4), two Zn^2+^ cations (Zn1 and Zn2), and two water molecules. The geometry around each Zn(II) ion is roughly square pyramidal (SQP); however, the degree of trigonality (τ) is 0.458 for Zn1 and 0.326 for Zn2, indicating a strong shift to trigonal bipyramidal (TBP). The τ value for ideal polyhedrons is τ = 0 for SQP and τ = 1 for TBP [31]. The basal plane of each polyhedron is formed by two oxygen and two nitrogen atoms (N_2_O_2_). The chelating glycine-ligands are arranged in the *trans* configuration. According to bond angles around the Zn cations, each basal plane of SQP is tetrahedrally distorted [32]: two *trans* oriented oxygen atoms lie above the best least-squares plane (N_2_O_2_), whereas two *trans* oriented nitrogen atoms are below it. The zinc cations sit 0.5 Å above the best plane toward the apical atom (Table S2). Interestingly, the apical bonds Zn1−O8 and Zn2−O6 are the shortest bonds observed around metal cations. The dihedral angle between the best least-squares basal-planes of Zn1 and Zn2 polyhedrons is 54.7(1)°. Bond lengths for the Zn(II) coordination sphere are given in Table 2.

Four independent glycine moieties differ each other in molecular conformation, as confirmed by the N−C−C−O torsion angle: ∠O1−C1−C2−N1 = 15.7(2)° (Gly-1); ∠O3−C3−C4−N2 = 4.8(2)° (Gly-2); ∠O5−C5−C6−N3 = 21.4(2)° (Gly-3); and ∠O7−C7−C8−N4 = 1.4(2)° (Gly-4). The bidentate coordination of the glycine ligands around the Zn atoms results in the formation of five-membered chelate rings adopting an envelope (Zn1/O1/C1/C2/N1 and Zn2/O7/C7/C8/N4) or twisted conformation (Zn1/O3/C3/C4/N2 and Zn2/O5/C5/C6/N3) according to asymmetry parameters [33]. (Table S1). The dihedral angle between the best least-squares planes of the chelate rings is 30.5(1)° around Zn1 and 24.7(1)° around Zn2.

Complex (**2**) exhibits a two-dimensional polymeric structure. The infinite (1 0 0) coordination sheets are separated by layers built from water molecules (Figure S1). Both types of structural layers are connected to each other via hydrogen bonds (Table S3).

A careful inspection of the packing within the coordination sheets revealed that two additional Zn···O interactions exist, Zn1···O4(*x*, 3/2−*y*, −½ + *z*) and Zn2···O2(*x*, *y*, 1 + *z*); their distances are much longer than those of the Zn−O bonds within the first Zn-coordination sphere, but are still shorter than the corresponding van der Waals radii of 3.77Å (Table 2). It is possible that these weak interactions complement the coordination geometries around each Zn(II) atom, thus significantly elongating the octahedral ones. It appears that all carboxylic oxygen atoms are involved in the coordination scheme; however, the Zn−O contacts have a different character (Figure 1).

#### 2.2.2. A Single-Crystal Structure of 3

Complex (**3**) crystallizes in a tetragonal system with the metal (Zn1) located on a twofold axis; the asymmetric unit consists of one histidine moiety, in the form of a monoanion, and two water molecules. As illustrated in Figure 1, the zinc cation is coordinated independently with two N atoms of the histidine moiety, thus giving a coordination mode without oxygen atoms, which is different to those of the other presented structures of Zn(II) with deprotonated amino acids, Gly (**2**), L-Pro (**4**), and L-Met (**5**). Moreover, the coordinative Zn−N bonds are also the shortest observed (Table 3). The geometry around the Zn1 cation is distorted tetrahedral, with the angles varying from 97.79(18)° to 118.1(3)°. The six-membered chelate ring Zn1/N1/N2/C2/C3/C4 adopts a conformation between envelope (with the C2 atom being out-of-plane) and half-chair (a pseudo two-fold axis passing through the Zn1−N2 bond) (Table S1). The dihedral angle between the symmetrically-related chelate rings around Zn1 is 75.4(2)°.

The most interesting feature of (**3**) is the organization of complex molecules in the crystal lattice. In contrast to structures (**1**), (**4**), and (**5**), no infinite coordination motifs are observed. In (**3**), the intermolecular interaction N3−H3⋅⋅⋅O1 (3/2 − *x*, ½ + *y*, ¾ − *z*) is responsible for the formation of the 3D hydrogen-bonded network. Thus, each complex molecule is associated with four molecules forming a supramolecular structure. Additionally, the crystal packing of (**3**) is stabilized by a number of O−H···O and N−H···O interactions where the water molecules play the role of proton acceptor and/or proton donor. The geometries of these contacts are presented in Table S3.

Although the supramolecular structure of (**3**) can be organized by complex molecules of the Zn(II) complex, the presence of water molecules in this structure seems to be important. The packing index calculated for the entire structure (**3**) is 74.1; however, this value decreases to 60.2 for the water-free structure, clearly demonstrating that water molecules fill the space between the complex molecules in the supramolecular architecture. The Zn(II) complex with glycine (**2**) also crystallizes as hydrate, with respective packing indices of 79.6 and 71.8 for the entire and water-free structures, respectively. It shows that the infinite metallopolymeric motifs generate more closely-packed structures, which is additionally supported by the packing indices of 76.8 and 75.4, calculated for the crystal structures of (**4**) and (**5**), respectively.

#### 2.2.3. A Single-Crystal Structure of **4**

As shown in Figure 1, in the molecular structure of (**4**), the Zn(II) is pentacoordinated. The Addison τ parameter for the zinc polyhedron has a value of 0.75, which indicates that the coordination geometry is closer to trigonal–bipyramidal (TBP, τ = 1) than square–pyramidal (SQP, τ = 0). The equatorial plane consists of O3, N1, and N2(−*x*, ½ + *y*, 1 − *z*) atoms; the zinc cation appears to rest on it, since it is displaced only by 0.023(4)Å towards the apical atom O1 (Table S2). The widest angle of 173.37(4)° is observed between atoms O1 and O4(−*x*, ½ + *y*, 1 − *z*) indicating the opposite peaks of the bipyramid. The Zn−O4 bond is the longest observed within the polyhedron (Table 4). Two independent L-proline moieties form five-membered chelate rings oriented in a *trans* configuration around a metal center. Their best planes form a dihedral angle of 65.3(1)°. The conformation of chelate rings, Zn1/O1/C1/C2/N1 and Zn1/O4^i^/C6^i^/C7^i^/N2^i^ (i: −*x*, ½ + *y*, 1 − *z*), can be described as twisted and envelope, respectively (Table S1).

The inspection of the crystal packing of (**4**) revealed metallopolymeric chains running along *b* axis (see Figure S2). In contrast to other investigated structures, only two hydrogen bonds exist in crystal lattice: an intermolecular interaction, N1−H1A···O1(*x*, 1 + *y*, *z*), is observed within molecules of polymeric chain, whereas a contact, N2−H2···O2(*x*, *y*, 1 + *z*), links the neighboring chain-motifs along the [001] direction.

#### 2.2.4. A Single-Crystal Structure of **5**

Although the crystal structure of (**5**) previously reported by Wilson et al. [29] is in good agreement with our determination (unit cell constants: *a* and *c* are swapped), we decided to use our low-temperature data for a more consistent comparison between investigated structures of all four Zn(II) complexes and Hirshfeld surface analysis.

The coordination geometry around Zn is a distorted octahedral with four short and two long bonds (Table 5). The basal plane of the octahedron is formed by two nitrogen (N1, N3) and two oxygen (O1, O3) atoms from two independent methionine moieties arranged in a *trans*-fashion. The RMS deviation of the fitted atoms from the best plane is 0.0672. The axial sites are occupied by oxygen atoms (O2, O4) from two other molecules. Thus, each Zn^2+^ cation is bonded with four methionine moieties, whereas each methionine monoanion is associated with two Zn(II) centers (Figure 1).

Two independent methionine moieties display different molecular conformations, as confirmed by corresponding torsion angles of ∠N1−C2−C3−C4 = 175.4(4)° and ∠N2−C7−C8−C9 = −54.7(5)°.

There are two five-membered chelate rings formed by methionine ligands: ring Zn1/O1/C1/C2/N1 adopts a conformation between envelope (with the C2 atom being out-of-plane) and twisted (pseudo two-fold axis passing through the O1−C1 bond), while ring Zn1/O3/C6/C7/N2 adopts an envelope conformation, with the C7 atom being out-of-plane (Table S1). The interplanar angle is 13.6(2)°.

The coordination polymer forms an infinite two-dimensional sheet framework lying parallel to the (001) plane (Figure S3). The thickness of each separate layer is equal to the *c* unit cell constant. The side-chain alkyl groups of methionine ligands are directed toward the outside layer.

### 2.3. Hirshfeld Surface Analysis of the Zn^2+^ Centre

**Figure 2:**
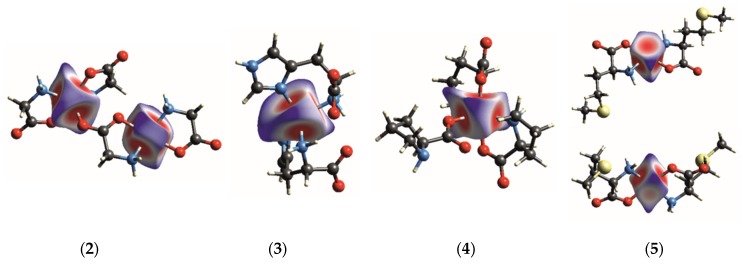
The Hirshfeld surface of the Zn^2+^ center in (**2**), (**3**), (**4**), and (**5**) with a normalized contact distance (*d_norm_*) mapped on it. The parameter *d*_norm_ ranges from −0.61 to 1.14 for Zn1 and from −0.62 to 1.12 for Zn2 (**2**); from −0.64 to 1.42 (**3**); from −0.62 to 1.26 (**4**), and from −0.59 to 0.90 (**5**).

The structural investigations of Zn(II) complexes with glycine (**2**), L-histidine (**3**), L-proline (**4**), and L-methionine (**5**) were extended by Hirshfeld surface analysis of the metallic center using CrystalExlorer [34,35]. Such an approach has been stressed in recent studies (see: Pinto et al.) [36]. Figure 2 presents the Hirshfeld surface (HS) of the Zn^2+^ center with the normalized contact distance (*d*_norm_) encoded on it. The geometrical *d*_norm_ function is defined as the sum of the distances of any surface point to the nearest atom outside (*d*_e_) and inside (*d*_i_) the surface, with both components normalized by the van der Waals radii of the atoms ($r_{i}^{vdW}$ and $r_{e}^{vdW}$, respectively) [37]. The minimum and maximum values of a geometrical function are presented as red and blue, respectively. Geometrical properties mapped on the Hirshfeld surface of the metal centers can be applied to rationalize coordination bonds.

As shown in Figure 2, all investigated surfaces differ in shape, indicating that the polyhedrons display different coordinations. The coordination bonds Zn−O and Zn-N are mostly drawn as very similar bright regions perpendicular to the bond direction, indicating their comparable strength. It is also evident that the Zn−O interaction completing the octahedral geometry in (**5**) (bottom drawing) is much weaker than those forming the square pyramid, but is still observed on the HS. In contrast, the long-range interactions observed in (**2**) are not visible on the HS. Both the HS shapes determined for Zn1 and Zn2 and its positive *d*_norm_ values indicate that Zn1···O4 and Zn2···O2 interactions are very weak van der Waals contacts.

**Figure 3:**
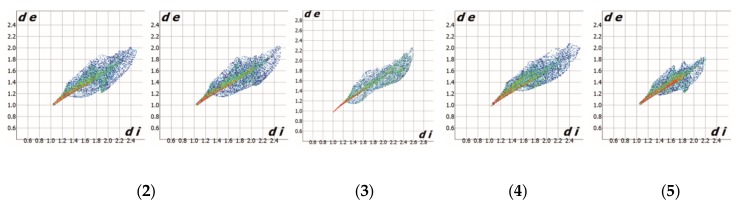
Fingerprint plot for the Hirshfeld surface of the metallic center of (**2**), (**3**), (**4**), and (**5**).

**Figure 4:**
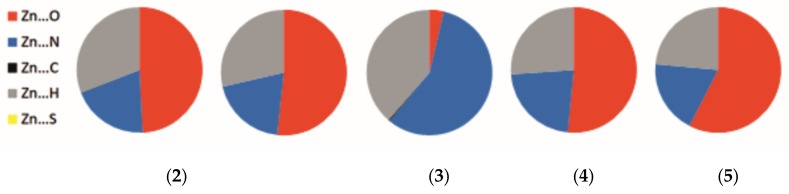
The percentage contribution of contacts of the Zn^2 +^ in complexes (**2**), (**3**), (**4**), and (**5).**

Figure 3 presents the fingerprint plot (two-dimensional histograms of *d*_i_ vs. *d*_e_) [38] for the Hirshfeld surface of the metallic center in (**2**), (**3**), (**4**) and (**5**). Such a plot generated for the Zn^2+^ cation reveals not only general but also very subtle differences in interaction schemes around metal. The overall form is reminiscent of a leaf shape. The central “leaf nerve” displays the shortest metal–ligand contacts (mainly red and green dots starting at 1Å of *d*_i_ vs. *d*_e_), which dominate in structure (**5**), demonstrating the smallest spread of the weakest interactions (*d*_i_ ≈ 2.2Å and *d*_e_ ≈ 1.8Å). A breakdown of each fingerprint plot for the metal into individual contacts is shown in Figure 4; corresponding fingerprint plots reduced to a single type of contact with Zn(II) are presented in Figure S4. It is clearly seen that structure (**3**) has a completely different interaction scheme regarding the metal center than the others. While the Zn···N (57.3%) and Zn···H (38.6%) contacts cover almost the entire coordination sphere in (**3**), the Zn···O contacts clearly dominate for the remaining structures (49–57%).

### 2.4. Antibacterial Activity

**Figure 5:**
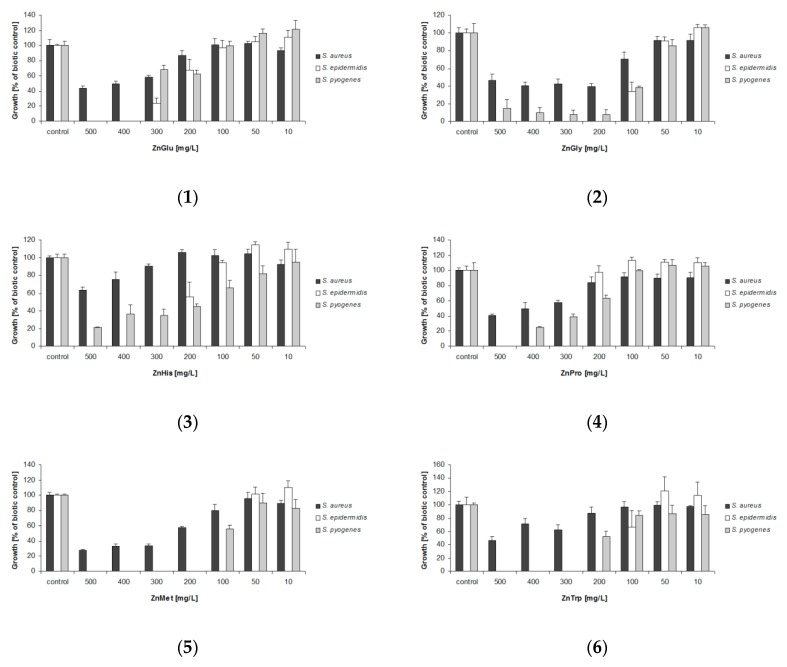

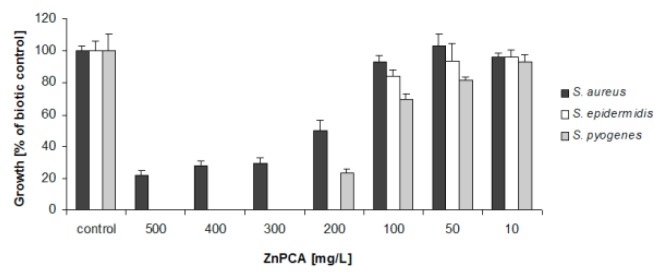
The antibacterial properties of zinc(II)-amino acid complexes (**1**–**6**) and the ZnPCA reference compound.

The antibacterial activity of the six synthesized complexes toward selected Gram-positive (Table 6, Figure 5) and Gram-negative (Figure S5) bacteria was determined and compared with that of zinc 2-pirrolidine 5-carboxylate (ZnPCA).

The results revealed that one of the tested complexes demonstrated similar activity to ZnPCA [20,39], and other two demonstrate better antibacterial properties.

Complex (**5**), ZnMet, possesses higher antimicrobial properties than the currently-used standard. The addition of 200 mg/L ZnMet to samples limited *S. aureus* growth by 45% (Figure 5); in addition, its minimum inhibitory concentration (MIC) values were found to be 100 mg/L for *S. epidermidis* and 200 mg/L for *S. pyogenes*, both of which were lower than those for ZnPCA (Table 6). Complex (**5**) also exhibited better antibacterial activity toward *E. coli* than ZnPCA (Figure S5). At the highest tested concentration, 60% bacterial growth inhibition was observed in relation to biotic control.

Complex (**2**), ZnGly, also demonstrated greater antibacterial activity than ZnPCA. The addition of 200 mg/L ZnGly to the cultures of *S. aureus* and *S. pyogenes* limited growth of bacteria by 60% and 90%, respectively (Figure 5). In analogous samples containing ZnPCA, *S. aureus* tolerance was 50% and growth inhibition of *S. pyogenes* reached the value of 75% (Figure 5). Complex (**2**) also exhibited stronger antimicrobial activity toward *P. aeruginosa* than ZnPCA (Figure S5).

Complex (**6**), ZnTrp, demonstrated comparable antimicrobial activity to ZnPCA, with both compounds demonstrating MIC values of 200 mg/L for *S. epidermidis* and 300 mg/L for *S. pyogenes* (Table 6).

The decreasing effectiveness of existing antibiotics and rapid growth of drug resistance among a variety of bacteria have added impetus to the search for newer, more effective antimicrobial compounds. Literature data indicate that some transition metal complexes of amino acid derivatives may exhibit good antimicrobial properties [24,40,41]. Our findings confirm that zinc–amino acid complexes exhibit antimicrobial activity against Gram-positive and Gram-negative bacteria, and that two of them were found to possess better antibacterial properties than ZnPCA, a commonly-used antiseptic agent in cosmetic products. Premlata et al. [42] also note that mixed zinc(II) complexes with 2-substituted benzothiazoles and amino acids are more active than tested standards.

Our findings revealed that in samples containing 500 mg/L of complexes, growth inhibition of tested Gram-negative microorganisms was in range from 20% to 80%. (Figure S5). The tested compounds showed higher antimicrobial activity toward Gram-positive than Gram-negative bacteria. Similar results were received by Aiyelabola et al. [43] who studied the antimicrobial properties of coordination compounds of aspartic acid. Similarly, copper and cobalt amino acids complexes also exhibited lower antibacterial activity toward Gram-negative bacteria [44]. Such differences in susceptibility of Gram-positive and Gram-negative bacteria to the tested complexes may be associated with differences in the structure of the cell membranes.

## 3. Materials and Methods

### 3.1. Chemicals and Apparatus

L-Glu, Gly, L-His, L-Met, L-Pro, and L-Trp were purchased from Alfa Aesar, ZnCl_2_ from Sigma-Aldrich and NaOH from POCH (Poland).

^1^H NMR spectra were recorded on a Bruker Avance III 600 MHz spectrometer using D_2_O or DMSO-*d*_6_ as the solvent. Elemental analyses (C, H, and N) were performed with a Perkin-Elmer 2400 analyzer.

### 3.2. Synthesis and Characterization of the Zn(II) Complexes (**1**–**6**)

#### 3.2.1. Preparation of Zn(OH)_2_

In this step, 44 mL of 1M NaOH (0.0440 mol) were added to the zinc chloride (3 g, 0.0220 mol) and mixed. The white solid immediately precipitated. The reaction mixture was left for about one hour on a magnetic stirrer and mixed intensively. The precipitate was then filtered off under reduced pressure, washed with cold water, and dried on air overnight.

#### 3.2.2. Synthesis of the Zn(II) Complexes (**1**–**4**)

Zinc hydroxide (1 mmol, 0.0994 g) was suspended in approximately 15 mL of distilled water; following this, a clear aqueous solution (approximately 10 mL) of a corresponding amino acid (2 mmol) (Glu (0.2942 g), Gly (0.1502 g), His (0.3104 g) and Pro (0.2302 g)) was added to the suspension. The reaction mixture was stirred at 50 °C for several minutes until the suspension was dissolved, and then left stirring for two hours. Any white solid, if it appeared, was filtered off and discarded. The remaining clear solution was evaporated to one-third of the initial volume and allowed to crystallize. After one to three days, the precipitated white solid product was washed with anhydrous diethyl ether and air-dried.

Zn(II) complex of L-glutamic acid. [Zn(Glu)_2_]·H_2_O (**1**): MW = 375.64; yield: 0.285 g (76%). Anal. Calcd for C_10_H_18_N_2_O_9_Zn ([Zn(Glu)_2_]·H_2_O): C, 31.97; H, 4.83; N, 7.46%. Found: C, 32.02; H, 5.02; N, 7.48%. ^1^H NMR (600 MHz, D_2_O): δ 1.95−2.07 (m, 4H, 2 x CH_2_(β)), 2,30–2,38 (m, 4H, 2 x CH_2_(γ)), 3,66–3,68 (m, 2H, 2 x CH(α)).

Zn(II) complex of glycine. [Zn(Gly)_2_] (**2**): MW = 213.50; yield: 0.181 g (85%). Anal. Calcd for C_4_H_8_N_2_O_4_Zn ([Zn(Gly)_2_]): C, 22.50; H, 3.75; N, 13.12%. Found: C, 22.51; H, 4.29; N, 12.97%. ^1^H NMR (600 MHz, D_2_O): δ 3.23 (s, 4H, 2 x CH_2_).

Zn(II) complex of L-histidine. [Zn(His)_2_]·2H_2_O (**3**): MW = 409.72; yield: 0.332 g (81%). Anal. Calcd for C_12_H_20_N_6_O_6_Zn ([Zn(His)_2_]·2H_2_O): C, 35,18; H, 4.92; N, 20.52%. Found: C, 35.13; H, 4.58; N, 20.53%. ^1^H NMR (600 MHz, D_2_O): δ 2.95–2.98 (m, 2H, 2 x CH_2_(6′)), 3.09–3.13 (dd, 2H, 2 x CH_2_(6′’)), 3.87–3.89 (dd, 2H, 2 x CH(7)), 6.93 (s, 2H, 2 x H(5)), 7.65 (s, 2H, 2 x H(2)).

Zn(II) complex of L-proline. [Zn(Pro)_2_] (**4**): MW = 293.63; yield: 0.200 g (68%). Anal. Calcd for C_10_H_16_N_2_O_4_Zn ([Zn(Pro)_2_]): C, 40.90; H, 5.49; N, 9.54%. Found: C, 40.91; H, 7.21; N, 9.55%. ^1^H NMR (600 MHz, D_2_O): δ 1.81 (m, 4H, 2 x CH_2_(4)), 2.19 (m, 4H, 2 x CH_2_(3)), 3.13 (m, 4H, 2 x CH_2_(5)), 3.86 (m, 2H, 2 x CH(2)).

#### 3.2.3. Synthesis of the Zn(II) Complexes (**5**–**6**)

Zn(II) complex of L-methionine. [Zn(Met)_2_]·0.5H_2_O (**5**): 

Method I: An aqueous solution (approximately 20 mL) of methionine (2 mmol, 0.2984 g) was added to a suspension of Zn(OH)_2_ (1 mmol, 0.0994 g) in water (ca. 15 mL) and stirred for three hours at 50 °C. After this time a white solid product was filtered off and washed with cold water, diethyl ether and dried in air. MW = 370.80; yield: 0.237 g (64%).

Method II: Briefly, 2 mL of 1M solution of NaOH (2 mmol) were added to the solid methionine (2 mmol, 0.2984 g) and mixed until it dissolved. An aqueous solution (approximately 10 mL) of zinc chloride (1 mmol, 0.1364 g) was mixed with the previous solution. The white product was precipitated immediately. The mixture was stirred for around an hour at a room temperature, and then filtered and washed with cold water and diethyl ether. MW = 370.80; yield: 0.308 g (83%).

Anal. Calcd for C_10_H_21_N_2_O_4.5_S_2_Zn ([Zn(Met)_2_]·0.5H_2_O): C, 32.39; H, 5.71; N, 7.56%. Found: C, 31.99; H, 5.40; N, 7.48%. ^1^H NMR (600 MHz, D_2_O): δ 1.86–1.92 (m, 2H, 2 x1H of CH(β)), 2.02 (s, 6H, 2 x CH_3_), 2,05–2,11 (m, 2H, 2 x1H of CH(β)), 2.50–2.59 (m, 4H, 2 xCH_2_-S), 3.53–3.58 (m, 2H, 2 x C*H*(α)).

Zn(II) complex of L-tryptophan. [Zn(Trp)_2_] (**6**): Briefly, solid tryptophan (2 mmol, 0.4085 g) was first dissolved in 2 mL of 1M NaOH (2 mmol). Following this, approximately 10 mL of aqueous zinc chloride solution (1 mmol, 0.1364 g) were added. A white product was precipitated immediately. The mixture was then stirred a further hour at room temperature, and subsequently filtered and washed with cold water and diethyl ether. MW = 471.81; yield: 0.425 g (90%). Anal. Calcd for C_22_H_22_N_4_O_4_Zn ([Zn(Trp)_2_]): C, 56.00; H, 4.70; N, 11.88%. Found: C, 55.87; H, 4.43; N, 11.89%. ^1^H NMR (600 MHz, DMSO): δ 2.87–2.92 (m, 2H, 2 x C*H*_2_(β’)), 3.32–3.35 (m, 2H, 2 x C*H*_2_(β’’)), 3.47–3.48 (m, 2H, 2 x C*H*(α)), 6.94–6.97 (m, 2H, 2 x *H*(3)(indol)), 7.05–7.07 (m, 2H, 2 x *H*(4)(indol)), 7.30 (s, 2H, 2 x *H*(7)(indol)), 7.35–7.37 (d, 2H, 2 x *H*(5)(indol)), 7.55–7.57 (d, 2H, 2 x *H*(2)(indol)), 10.96 (s, 2H, 2 x N*H*(1)(indol)) (Figure S6).

### 3.3. X-ray Diffraction Studies

Single crystal X-ray data were collected on a micro-focus SuperNova diffractometer with an Atlas detector (**3**, **4**, **5**) and Xcalibur Sapphire detector (**2**), all using MoKα (CuKα was exceptionally used for **5**) and ω scan. The measurements were carried out at low temperature of 100(2)K. The X-ray data were corrected for absorption [45,46]. All structures were solved using SHELXT [47] and refined with SHELXL-2018/3 [48].

Generally, all (C)-H atoms were geometrically placed and constrained to their parent atoms as a rigid body. All other H atoms were located in a difference map and refined isotropically.

Structure (**3**) was refined as a two-component twin with BASF 0.0486. The H-atoms of the water molecules (H31W, H32W, H41W, H42W) were refined with the O−H distances restrained to 0.84Å (*U*_iso_ = 1.5 *U*_eq_ of atom O3W and O4W, respectively), whereas the N3−H3 distance was restrained to 0.86Å (*U*_iso_ = 1.2 *U*_eq_ of atom N3).

In (**4**), a distance DFIX-restraint of 0.86Å was used for N2−H2A bond length.

The crystallographic programs MERCURY [49] and PLATON [50] and were used for structure analysis and presentation of the results.

### 3.4. Antibacterial Activity Study

The antibacterial properties of complexes **1** to **6** were determined with respect to three Gram-positive strains, viz. *Staphylococcus aureus* ATCC 6538, *Staphylococcus epidermidis* ATCC 12,228, and *Streptococcus pyogenes* ATCC 19615, and two Gram-negative bacteria: *Escherichia coli* ATCC 25,992 and *Pseudomonas aeruginosa* 27853. The growth of microorganisms in the presence of the tested compounds was estimated by a modified broth microdilution method, in accordance with the Clinical and Laboratory Standards Institute (CLSI M07–18). The obtained results were presented as tolerance toward the studied complex (% of biotic control) or as minimum inhibitory concentration (MIC). The tested compounds were dissolved in water (**1**–**4**) or in DMSO (**5**–**6**).

## 4. Conclusions

Our investigations concerns bis(aminoacidate) zinc(II) complexes as potentially safe and effective antibacterial components for dermatological treatment. The combination of zinc, one of the most important trace elements, with proteinogenic amino acids afforded compounds expected to be non-toxic, well tolerable, and effective agents for skin diseases involving inflammation and irritation of the skin, such as ulcers and acne vulgaris.

The synthesis was designed in such a way that the final complexes did not contain any counter-ions, such as Cl^−^, NO_3_^−^ or SO_4_^2−^, that could cause skin irritations. The simplicity of the complexes’ composition is their advantage; they contain only zinc and aminoacidates ligands.

The research presents a thorough investigation of the crystal structures of zinc(II) complexes of selected amino acids. Single-crystal X-ray diffraction analysis found that compounds ZnGly (**2**) and ZnMet (**5**) have a 2D-coordination-layer architecture; ZnPro (**4**) is characterized by 1D-metallopolymeric chains and the complex molecules of ZnHis (**3**) are organized into a 3D hydrogen-bonded network. The Hirshfeld surface analysis for the metallic center showed that all Zn-surfaces differed in shape, as confirmed by their different coordination geometries: square–pyramidal in (**2**), tetrahedral in (**3**), trigonal–bipyramidal in (**4**), and octahedral in (**5**).

The antimicrobial analysis found two compounds, ZnGly (**2**) and ZnTrp (**6**), to exhibit promising antibacterial properties, and another one, ZnMet (**5**), to demonstrate greater antibacterial activity than the tested standard. All complexes exhibit better antimicrobial properties against Gram-positive bacteria than Gram-negative microorganisms.

Further antimicrobial studies and cytotoxicity tests on the zinc(II)–AA complexes will be the subject of our next study.

## Figures and Tables

**Table 1:** Crystallographic data for complexes of Zn(II) with glycine (**2**), L-histidine (**3**), L-proline (**4**), and L-methionine (**5**).

	(2)	(3)	(4)	(5)
Empirical Formula	C_8_H_16_N_4_O_8_Zn_2_⋅2(H_2_O)	C_12_H_16_N_6_O_4_Zn⋅4(H_2_O)	C_10_H_16_N_2_O_4_Zn	C_10_H_20_N_2_O_4_S_2_Zn
Formula Weight	463.02	445.74	293.62	361.77
Crystal System	Monoclinic	Tetragonal	Monoclinic	Monoclinic
Space Group	*P*2_1_/c	*P*4_3_2_1_2	*P*2_1_	*P*2_1_
*a* (Å)	15.0075(6)	7.5879(2)	9.3868(1)	9.3952(3)
*b* (Å)	10.4204(4)	31.1659(11)	5.5883(1)	5.0863(1)
*c* (Å)	9.7717(4)	7.5879(2)	10.6039(1)	15.4324(6)
α (°)	90.000	90.000	90.000	90.000
β (°)	92.646(3)	90.000	93.017(1)	107.657(4)
γ (°)	90.000	90.000	90.000	90.000
*V* (Å^3^)	1526.51(11)	1794.41(11)	555.47(1)	792.72(4)
*Z*	4	4	2	2
*T* (K)	100(2)	100(2)	100(2)	100(1)
*F*(000)	944	928	304	376
*D_x_* (g cm^−3^)	2.015	1.650	1.755	1.710
*μ* (mm^−1^)	3.20	1.43	2.22	5.32
Wavelength (Å)	0.71073	0.71073	0.71073	1.54184
*θ* range (°)	3.4–30.0	3.00–29.0	3.9–33.0	3.00–70.0
Measured Reflections	14323	17886	7230	4735
Unique Reflections	4407	2396	3310	2127
Observed Reflections [*I* > 2σ(*I*)]	3731	2341	3264	2093
Completeness to *θ*_max_ (%)	98.9	99.7	99.7	99.6
parameters/Restraints	265/0	145/5	162/2	190/1
*R* [*I* > 2σ(*I*)]	0.030	0.053	0.014	0.033
*wR* (all data)	0.084	0.113	0.039	0.087
*S*	1.04	1.24	1.08	1.04
Δ*ρ*_max_ (e Å^−3^)	2.17 *^a^*	1.06 *^b^*	0.36	0.63
Δ*ρ*_min_ (e Å^−3^)	−0.78 *^a^*	−1.05 *^b^*	−0.28	−0.94

^a^ The highest peak and deepest hole are close to the zinc atom, at 1.19 Å and 0.67 Å from Zn1, respectively. ^b^ The highest peak is 0.86 Å from Zn1, whereas the deepest hole is 1.75Å from N2.

**Table 2:** Bond lengths to the Zn(II) ions (Å) for (**2**).

Bond	Distance	Bond	Distance
Zn1−O1	2.1407(13)	Zn2−O5	2.1915(12)
Zn1−O3	2.1262(12)	Zn2−O7	2.1205(12)
Zn1−O8	2.0127(12)	Zn2−O6^i^	2.0026(12)
Zn1−N1	2.0321(15)	Zn2−N3	2.0257(15)
Zn1−N2	2.0320(16)	Zn2−N4	2.0224(15)
Zn1···O4 ^ii^	3.5650(13)	Zn2···O2 ^iii^	3.4259(13)

Symmetry code: (i) *x*, ½ − *y*, −½ + *z*; (ii) *x*, 3/2 − *y*, −½ + *z*; (iii) *x*, *y*, 1 + *z*.

**Table 3:** Bond lengths to Zn(II) ion (Å) for (**3**).

Bond	Distance
Zn1−N1	1.989(4)
Zn1−N1 ^i^	1.989(4)
Zn1−N2	2.026(4)
Zn1−N2 ^i^	2.026(4)

Symmetry code: (i) 1 − *y*, 1 − *x*, ½ − *z.*

**Table 4:** Bond lengths to the Zn(II) ion (Å) for (**4**).

Bond	Distance
Zn1−O1	2.094(1)
Zn1−O3	1.9937(9)
Zn1−O4 ^i^	2.1535(10)
Zn1−N1	2.0556(11)
Zn1−N2 ^i^	2.0559(9)

Symmetry code: (i) −*x*, ½ + *y*, 1−*z*.

**Table 5:** Bond lengths to the Zn(II) ion (Å) for (**5**).

Bond	Distance
Zn1−O1	2.051(3)
Zn1−O3	2.059(3)
Zn1−O4 ^i^	2.221(3)
Zn1−O2 ^iii^	2.656(3)
Zn1−N1	2.093(4)
Zn1−N2	2.064(4)

Symmetry code: (i) 1 − *x*, ½ + *y*, 1−*z*; (iii) −*x*, −½ + *y*, 1 − *z*.

**Table 6:** Minimum inhibitory concentration (MIC) values of Zn(II) complexes for Gram-positive bacteria.

Tested Compound	MIC (mg/L) *Staphylococcus epidermidis*	*Streptococcus pyogenes*
ZnGlu (**1**)	400	400
ZnGly (**2**)	200	ND *
ZnHis (**3**)	300	ND *
ZnPro (**4**)	300	500
ZnMet (**5**)	100	200
ZnTrp (**6**)	200	300
ZnPCA	200	300

MIC for *Staphylococcus aureus* >500 mg/L; * ND—not detected.

## Supplementary Materials

The following are available online at https://www.mdpi.com/1420-3049/25/4/951/s1. Table S1: The asymmetry parameters for selected rings of (**2**), (**3**), (**4**), and (**5**). Table S2: The least-squares planes and deviations from them for (**2**), (**4**), and (**5**). Table S3: Hydrogen-bonding geometries (Å,°) of Zn(II) complexes with glycine (**2**), L-histidine (**3**), L-proline (**4**), L-methionine (**5**). Figure S1: A part of the crystal structure showing a single coordination sheet of (**2**) (*a*). The crystal packing of (**2**), view along the *b* axis (*b*). Figure S2: A part of the crystal structure showing a single coordination chain of (**4**). For clarity the ring-fragment of the L-proline moiety is omitted (*a*). The crystal packing of (**2**), view along the *b* axis (*b*). Figure S3: A part of the crystal structure showing a single coordination sheet of (**5**). For a comparison to structure (**2**) the side-chain of L-methionine moieties is omitted (*a*). The crystal packing of (**2**), view along the *c* axis (*b*). Figure S4: Fingerprint plot for the Hirshfeld surface of the metallic center of (**2**), (**3**), (**4**), and (**5**) and corresponding plots reduced to a given contact type: Zn···O, Zn···N, and Zn···H. Figure S5: The antibacterial properties of zinc(II)-aminoacidate complexes (**1**–**6**) and the ZnPCA reference compound towards Gram-negative bacteria. Figure S6. Exemplary ^1^H NMR spectra of L- tryptophan (*a*) and its zinc(II) complex (**6**) (*b*). Appendix S1. Description of ^1^H NMR spectra of free amino acids: L-Glu, Gly, L-His, L-Pro, L-Met, L-Trp.
