# Supplementary material for: Zinc(II) Complexes with Amino Acids for Potential Use in Dermatology: Synthesis, Crystal Structures, and Antibacterial Activity
Source: Molecules. 2020 Feb 20;25(4):951. doi: 10.3390/molecules25040951 (PMC7070670; doi:10.3390/molecules25040951)
Supplement: Supplementary file 1 [file molecules-25-00951-s001.pdf]

Zinc(II) complexes of amino acids for potential use in dermatology: synthesis, crystal structures and antibacterial activity

Michał Abendrot [1], Lilianna Chęcińska [2], Joachim Kusz [3], Katarzyna Lisowska [4], Katarzyna Zawadzka [4], Aleksandra Felczak [4,*] and Urszula Kalinowska-Lis [1,*]

[1] Department of Cosmetic Raw Materials Chemistry, Faculty of Pharmacy, Medical University of Lodz, Muszyńskiego 1, 90-151 Łódź, Poland.
[2] Faculty of Chemistry, University of Lodz, Pomorska 163/165, 90-236 Łódź, Poland
[3] Institute of Physics, University of Silesia, 75 Pułku Piechoty 1, 41-500 Chorzów, Poland
[4] Department of Industrial Microbiology and Biotechnology, Faculty of Biology and Environmental Protection, University of Lodz, 12/16 Banacha Street, 90-237 Łódź, Poland
* Correspondence: aleksandra.felczak@biol.uni.lodz.pl (A.F.), urszula.kalinowska-lis@umed.lodz.pl (U.K.-L.)

SUPPLEMENTARY MATERIALS

Table S1. The asymmetry parameters for selected rings of (**2**), (**3**), (**4**) and (**5**).

Compound	Ring	Asymmetry parameters	Conformation
(**2**)	Zn1/O1/C1/C2/N1	**ΔC_s(N1)=4.9(2)°**<br>ΔC_2(N1–C2)=8.4(2)°	**E**/T
	Zn1/O3/C3/C4/N2	ΔC_s(Zn1)=5.6(2)°<br>**ΔC_2(Zn1–N2)=4.8(2)°**	E/**T**
	Zn2/O5/C5/C6/N3	ΔC_s(N3)=10.4(2)°<br>**ΔC_2(N3–C6)=3.5(2)°**	E/**T**
	Zn2/O7/C7/C8/N4	**ΔC_s(Zn2)=1.9(2)°**<br>ΔC_2(Zn2–N4)=8.6(2)°	**E**/T
(**3**)	Zn1/N1/N2/C2/C3/C4	**ΔC_s(C2)=13.0(4)°**<br>ΔC_2(Zn1–N2)=15.2(5)°	**E/H**
(**4**)	Zn1/O1/C1/C2/N1	ΔC_s(Zn1)=2.6(2)°<br>ΔC_s(O1)=3.0(2)°<br>**ΔC_2(Zn1–O1)=1.9(2)°**	E/**T**
	Zn1/O4^i/C6^i/C7^i/N2^i	**ΔC_s(C6)=4.4(2)°**<br>ΔC_2(O4^i–C6^i)=5.4(2)°	**E**/T
	N1/C2/C3/C4/C5	ΔC_s(C4)=3.2(2)°	**E**
	N2/C7/C8/C9/C10	ΔC_s(C9)=7.1(2)°	**E**
(**5**)	Zn1/O1/C1/C2/N1	ΔC_s(C2)=7.6(5)°<br>ΔC_2(O4–C6)=7.8(5)°	**E/T**
	Zn1/O3/C6/C7/N2	ΔC_s(C7)=3.3(5)°	**E**

Symmetry code for (**4**) (i) −*x*, ½+*y*, 1−*z*.

Table S2. The least-squares planes and deviations from them (calculated by SHELXL using MPLA instruction) for (**2**), (**4**) and (**5**).

(2)- basal plane of square-pyramids of Zn1 and Zn2

```
Least-squares planes (x,y,z in crystal coordinates) and deviations from them
(* indicates atom used to define plane)

  9.4925 (0.0070) x +  4.3116 (0.0052) y -  6.6767 (0.0033) z =  3.3603 (0.0033)

 * -0.2339 (0.0008)  O1
 *  0.2203 (0.0007)  N1
 * -0.2251 (0.0007)  O3
 *  0.2386 (0.0008)  N2
   -0.5530 (0.0007)  Zn1

 Rms deviation of fitted atoms =   0.2296



 - 9.6641 (0.0068) x +  5.2393 (0.0047) y +  5.9193 (0.0034) z =  3.9534 (0.0046)

 Angle to previous plane (with approximate esd) = 54.771 ( 0.041 )

 * -0.1572 (0.0007)  O5
 *  0.1531 (0.0007)  N3
 * -0.1549 (0.0007)  O7
 *  0.1590 (0.0007)  N4
   -0.5151 (0.0007)  Zn2

 Rms deviation of fitted atoms =   0.1561
```

(4) – equatorial plane of trigonal bipyramid of Zn1

```
Least-squares planes (x,y,z in crystal coordinates) and deviations from them
 (* indicates atom used to define plane)

 - 1.4143 (0.0045) x +  4.1727 (0.0017) y +  6.9447 (0.0035) z =  5.0128 (0.0010)

 *  0.0000 (0.0000)  N1
 *  0.0000 (0.0000)  O3
 *  0.0000 (0.0000)  N2_$2
   -0.0231 (0.0007)  Zn1

 Rms deviation of fitted atoms =   0.0000
```

(5) – equatorial plane of octahedron of Zn1

```
Least-squares planes (x,y,z in crystal coordinates) and deviations from them
 (* indicates atom used to define plane)

  5.0688 (0.0080) x +  2.5020 (0.0057) y +  7.5234 (0.0169) z =  5.8953 (0.0064)

 *  0.0682 (0.0017)  O1
 * -0.0670 (0.0017)  N1
 *  0.0661 (0.0017)  O3
 * -0.0673 (0.0018)  N2
    0.2032 (0.0019)  Zn1

 Rms deviation of fitted atoms =   0.0672
```

Table S3. Hydrogen-bonding geometries (Å,°) of Zn(II) complexes with glycine (**2**), L-histidine (**3**), L-proline (**4**), L-methionine (**5**).

Comp.	H-bond	D–H	H···A	D···A	D–H···A
(**2**)	N1–H1A···O10W^{ii}	0.86(3)	2.05(3)	2.900(2)	169(2)
	N1–H1B···O3^{ii}	0.89(2)	2.35(2)	3.060(2)	136(2)
	N1–H1B···O7^{ii}	0.89(2)	2.45(2)	3.157(2)	136(2)
	N2–H2A···O5^{i}	0.79(3)	2.55(3)	3.258(2)	149(2)
	N2–H2B···O4^{ii}	0.97(3)	2.10(3)	3.007(2)	155(2)
	N3–H3A···O9W^{v}	0.91(2)	2.03(2)	2.937(2)	171(2)
	N3–H3B···O3^{vi}	0.92(2)	2.23(2)	3.054(2)	148(2)
	N4–H4A···O1^{iv}	0.83(3)	2.23(3)	2.992(2)	152(2)
	N4–H4B···O2^{iii}	0.93(3)	2.12(3)	2.975(2)	151(2)
	O9W–H9A···O4^{v}	0.85(3)	1.89(3)	2.723(2)	170(3)
	O9W–H9B···O6^{vii}	0.80(3)	2.05(3)	2.845(2)	173(3)
	O10W–H10A···O8^{viii}	0.83(3)	2.00(3)	2.827(2)	173(3)
	O10W–H10B···O2^{iii}	0.81(3)	1.93(3)	2.732(2)	169(3)
(**3**)	N1–H1A···O4^{ii}	0.90(7)	2.21(7)	3.034(6)	153(6)
	N1–H1B···O2^{i}	0.86(7)	2.16(7)	2.920(6)	146(6)
	N3–H3···O1^{iii}	0.87(3)	1.96(3)	2.786(5)	159(6)
	O3–H31W···O4	0.84(1)	1.95(2)	2.781(6)	167(7)
	O3–H32W···O2^{iv}	0.84(1)	1.95(1)	2.780(6)	173(7)(
	O4–H41W···O1^{v}	0.84(1)	1.90(2)	2.727(5)	170(8)
	O4–H42W···O3^{vi}	0.84(1)	1.96(2)	2.780(6)	164(7)
(**4**)	N1–H1A···O1^{iii}	0.88(2)	2.23(2)	3.020(2)	150(2)
	N2–H2A···O2^{iv}	0.84(1)	2.11(2)	2.906(2)	159(2)
(**5**)	N1–H1A···O4^{ii}	0.91(6)	2.71(6)	3.425(5)	136(5)
	N1–H1B···O1^{v}	0.88(9)	2.19(9)	2.944(5)	143(7)
	N1–H1B···O1^{iii}	0.88(9)	2.37(8)	3.007(5)	129(7)
	N2–H2A···O3^{vi}	0.87(6)	2.34(6)	3.145(5)	155(5)
	N2–H2A···O3^{i}	0.87(6)	2.33(5)	2.923(5)	125(5)
	N2–H2B···O2^{iv}	0.85(8)	2.61(8)	3.207(5)	128(6)

Symmetry codes: **2**: (i) *x*, ½−*y*, −½+*z*; (ii) *x*, 3/2−*y*, −½+*z*; (iii) *x*, *y*, 1+*z*; (iv) *x*, ½−*y*, ½+*z*; (v) 1−*x*, 1−*y*, 1−*z*; (vi) *x*, 3/2−*y*, ½+*z*; (vii) *x*, *y*, −1+*z*; (viii) −x, 1−*y*, 1−z.
Symmetry codes: **3**: (i) 1−*y*, 1−*x*, ½−*z*; (ii) ½+*x*, 3/2−*y*, ¼−*z*; (iii) 3/2−*x*, ½+*y*, ¾−*z*; (iv) ½−*y*, −½+*x*, −¼+*z*; (v) 1−*y*, 2−*x*, ½−*z*; (vi) −½+*x*, 3/2−*y*, ¼−*z*.
Symmetry codes: **4**: (iii) *x*, 1+*y*, *z*; (iv) *x*, *y*, 1+*z*.
Symmetry codes: **5**: (i) 1−*x*, ½+*y*, 1−z; (ii) 1−*x*, −½+*y*, 1−*z*; (iii) −*x*, −½+*y*, 1−z; (iv) −*x*, ½+*y*, 1−*z*; (v) *x*, −1+y, *z*; (vi) *x*, 1+*y*, *z*.

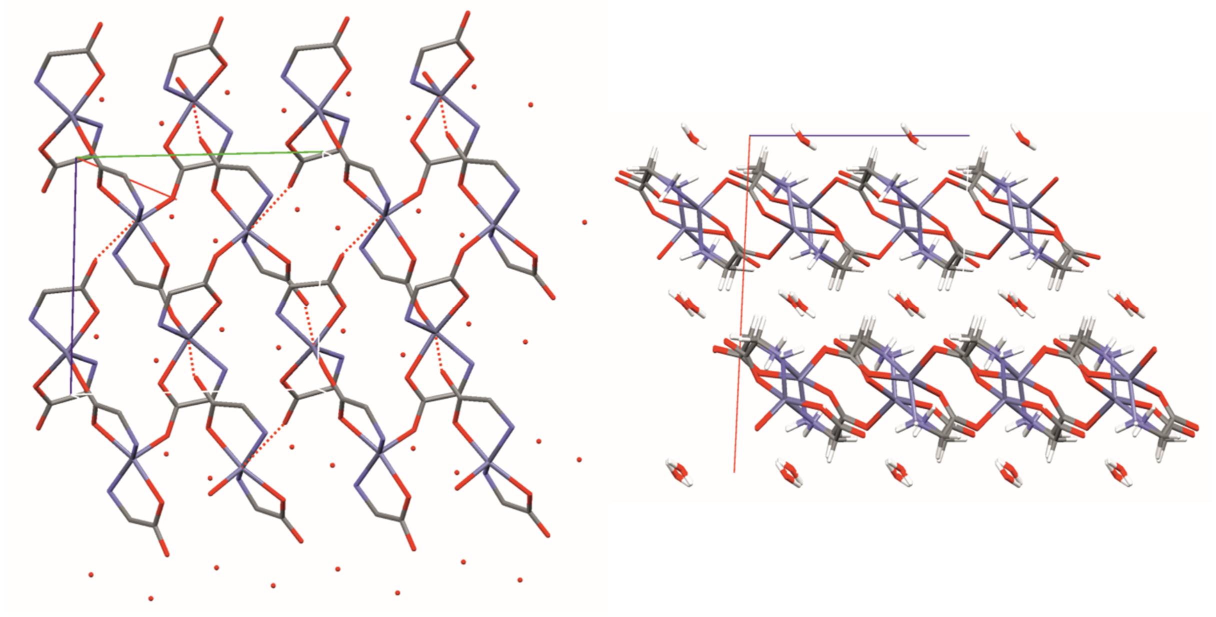

a *b*

Figure S1. A part of the crystal structure showing a single coordination sheet of (**2**) (*a*). The crystal packing of (**2**), view along *b* axis (*b*).

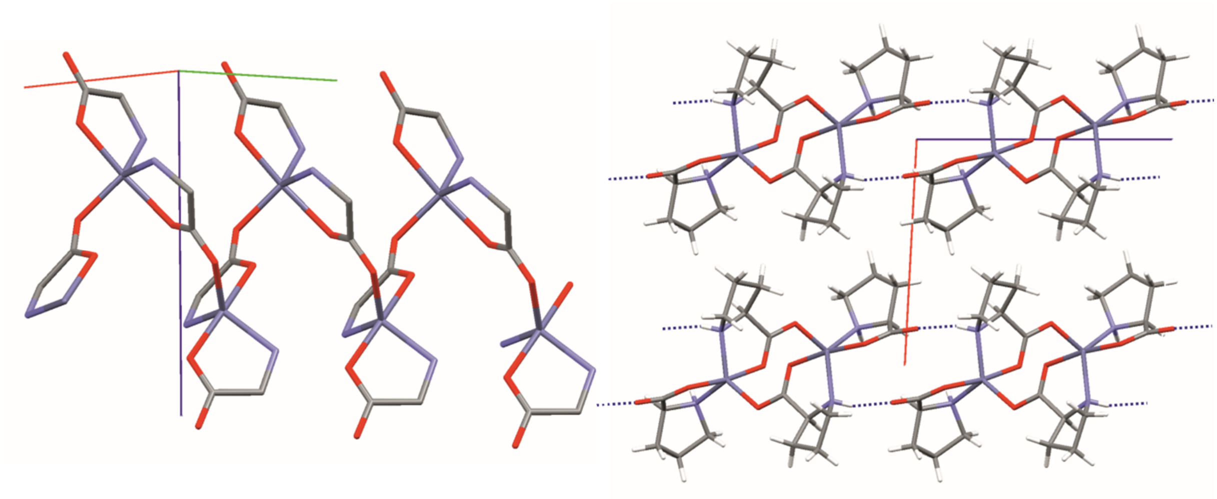

a *b*

Figure S2. A part of the crystal structure showing a single coordination chain of (**4**). For clarity reason the ring-fragment of L-proline moiety is omitted (*a*). The crystal packing of (**2**), view along *b* axis (*b*).

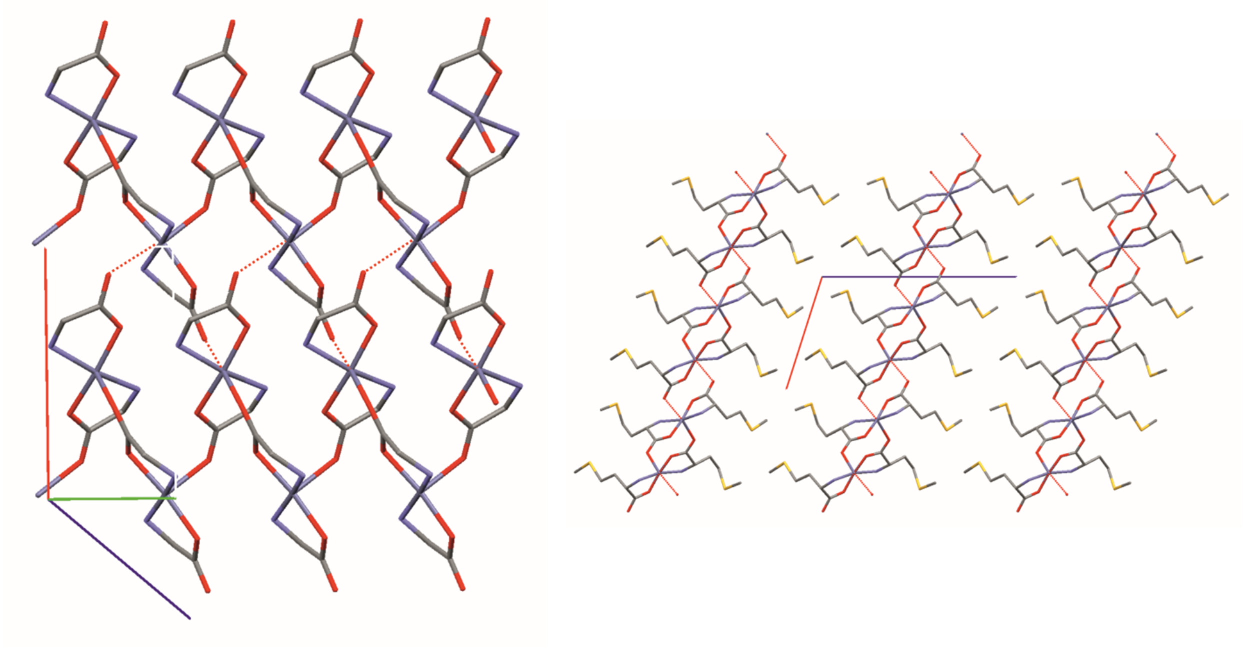

a *b*

Figure S3. A part of the crystal structure showing a single coordination sheet of (**5**) For a comparison reason to structure (**2**) the side-chain of L-methionine moieties is omitted (*a*). The crystal packing of (**2**), view along *c* axis (*b*).

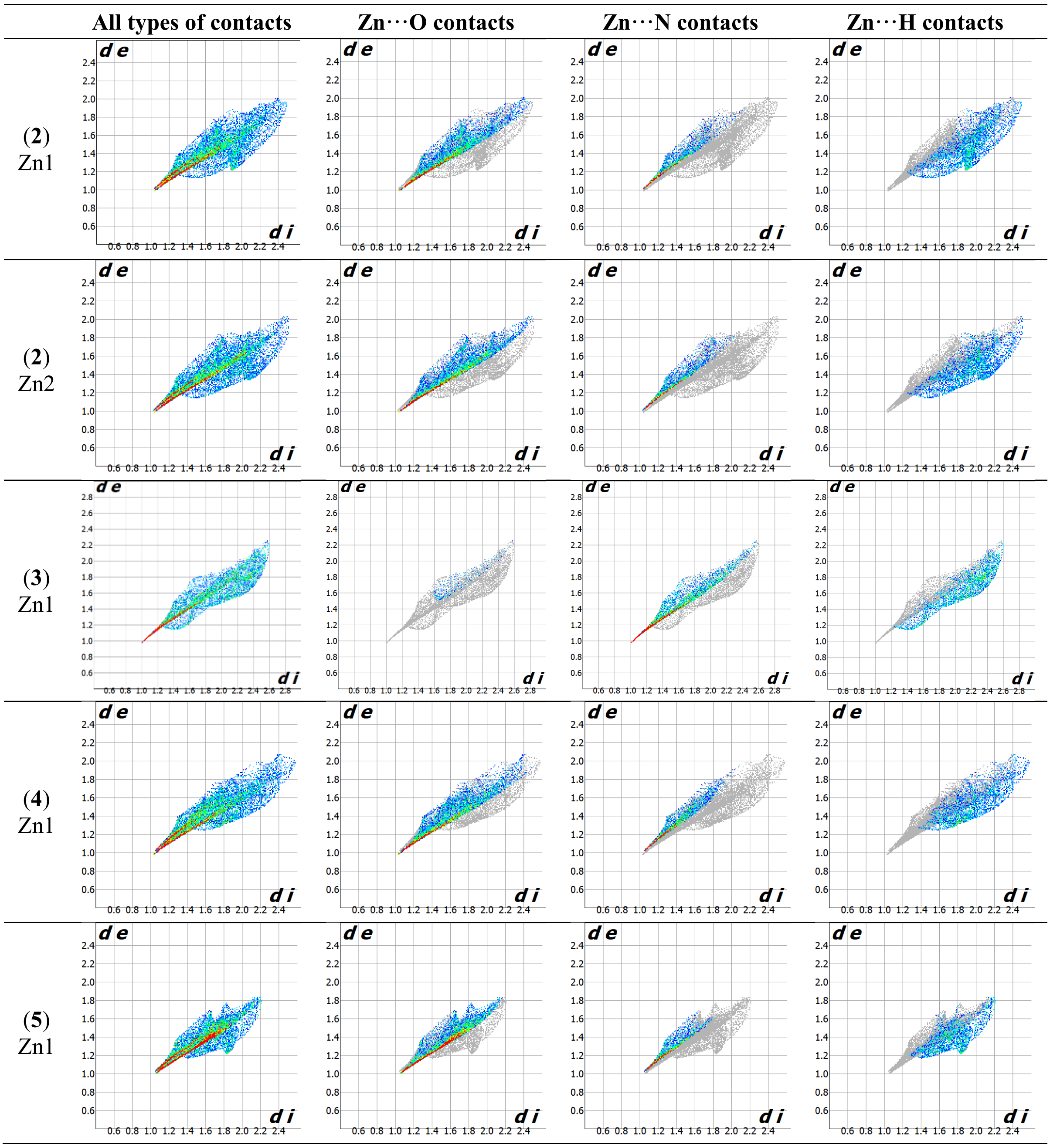

Figure S4. Fingerprint plot for the Hirshfeld surface of the metallic centre of (**2**), (**3**), (**4**) and (**5**) and corresponding plots reduced to a given contact type: Zn···O, Zn···N and Zn···H.

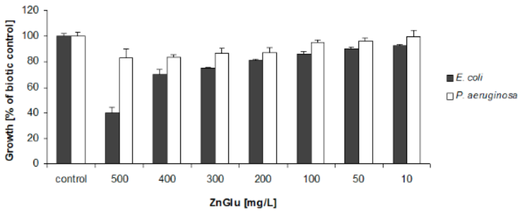

(1)

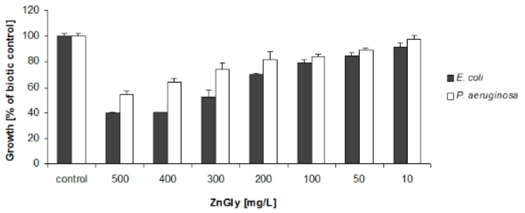

(2)

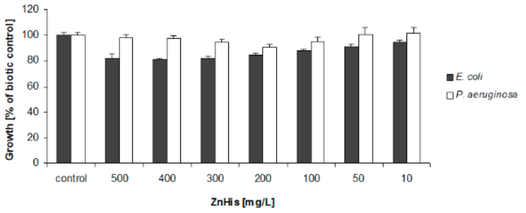

(3)

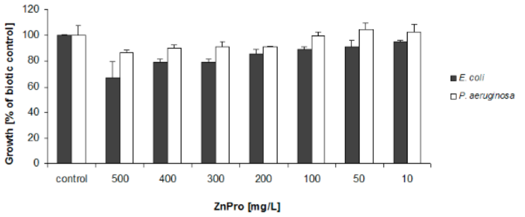

(4)

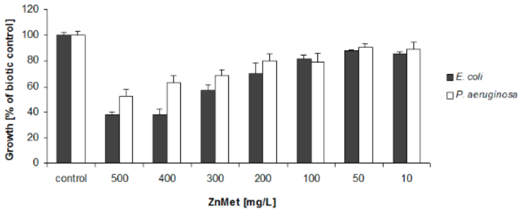

(5)

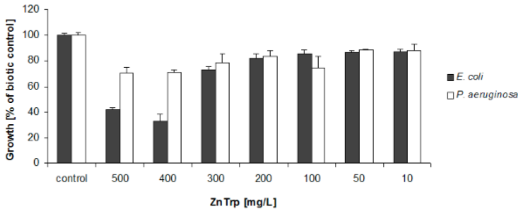

(6)

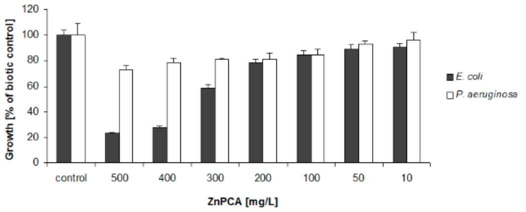

Figure S5. The antibacterial properties of zinc(II)- aminoacidate complexes (**1**-**6**) and ZnPCA reference compound towards Gram-negative bacteria.

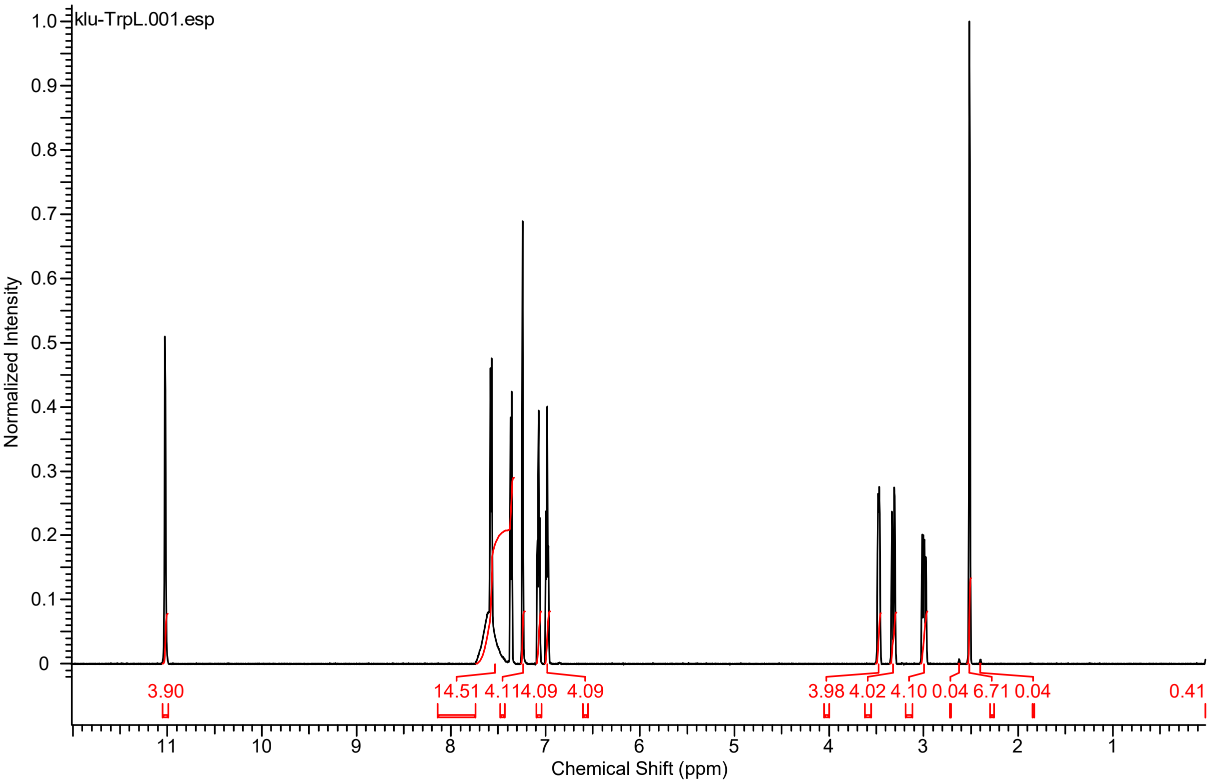

(a) ^{1}H NMR (DMSO) spectrum of L- tryptophan

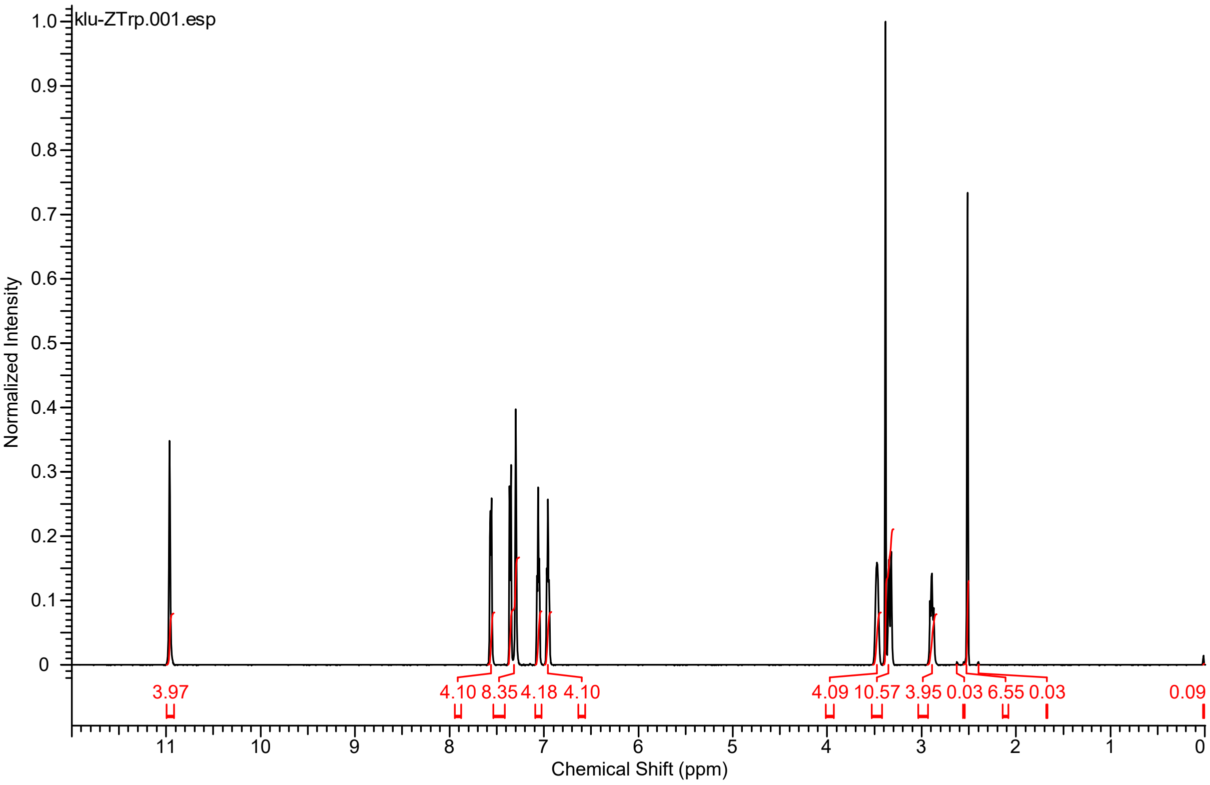

(b) ^{1}H NMR (DMSO) spectrum of zinc (II) complex of L- tryptophan (**6**)

Figure S6. Exemplary ^{1}H NMR spectra of L- tryptophan (*a*) and its zinc(II) complex (**6**) (*b*).

Appendix S1. Description of ^{1}H NMR spectra of free amino acids: L-Glu, Gly, L-His, L-Pro, L-Met, L-Trp,

L-Glutamic acid (for comparative purpose)

^{1}H NMR (600 MHz, D_2O): δ 2.01-2.10 (m, 2H, CH_2(β)) (Δ= -0.06), 2.42-2.49 (m, 4H, 2 x CH_2(γ)) (Δ= -0.12), 3.70-3.72 (m, 1H, CH(α)) (Δ= -0.04).

Glycine (for comparative purpose)

^{1}H NMR (600 MHz, D_2O): δ 3.53 (s, 1H, CH_2). (Δ= -0.30).

L-Histidine (for comparative purpose)

^{1}H NMR (600 MHz, D_2O): δ 2.95-2.98 (m, 1H, CH_2(6′)) (Δ=0), 3.06-3.10 (dd, H, CH_2(6′′)) (Δ=+0.03), 3.83-3.85 (dd, 1H, CH(7)) (Δ=+0.04), 6.90 (s, 1H, H(5)) (Δ=+0.03), 7.60 (s, 1H, H(2)) (Δ=+0.05).

L-Proline (for comparative purpose)

^{1}H NMR (600 MHz, D_2O): δ 1.84-1.96 (m, 3H, 2H of CH_2(4) (Δ= -0,03) and 1H of CH_2(3′)), 2.19-2.25 (m, 1H, CH_2(3′′)) (Δ=0), 3.18-3.23 (m, 1H, CH(5″)), 3.27-3.32 (m, 1H, CH(5′)) (Δ= -0,14), 3.98-4.01 (m, 1H, CH(2)) (Δ= -0,12).

L-Methionine (for comparative purpose)

^{1}H NMR (600 MHz, (D_2O): δ 2.01 (m, 3H, CH_3) (Δ= -0,01), 2,05-2,13 (m, 2H, CH_2) (Δ= -0.19 and Δ= -0,01), 2,50-2,55 (t, 2H, CH_2-S) (Δ= 0), 3,73-3,75 (dd, 1H, C*H*(α)) (Δ= -0,20).

L-Tryptophan (for comparative purpose)

^{1}H NMR (600 MHz, DMSO): δ 2.98-3.02 (m, 1H, CH_2(β′)) (Δ= -0,11), 3.31-3.35 (m, 1H, CH_2(β′′)) (Δ= 0,01), 3.47-3.38 (m, 1H, C*H*(α)) (Δ= 0), 6.97-7.00 (m, 1H, *H*(3)(indol)) (Δ= -0,03), 7.06-7.09 (m, 1H, *H*(4)(indol)) (Δ= -0,02), 7.24 (s, 1H, *H*(7)(indol)) (Δ= 0,06), 7.35-7.37 (d, 1H, *H*(5)(indol)) (Δ= 0), 7.52-7.58 (d, 1H, *H*(2)(indol)) (Δ= -0.02), 11.02 (s, 1H, N*H*(1)(indol)) (Δ= -0.06).

where Δ is a difference in values of chemical shifts of corresponding signals of free ligands and their Zn(II) complexes.
